# Pay Dispersion and Performance in Teams

**DOI:** 10.1371/journal.pone.0112631

**Published:** 2014-11-14

**Authors:** Alessandro Bucciol, Nicolai J. Foss, Marco Piovesan

**Affiliations:** 1 Department of Economics, University of Verona, Verona, Italy; 2 Department of Strategic Management and Globalization, Copenhagen Business School, Frederiksberg, Denmark; 3 Department of Economics, University of Copenhagen, Frederiksberg, Denmark; Institutes for Behavior Resources and Johns Hopkins University School of Medicine, United States of America

## Abstract

Extant research offers conflicting predictions about the effect of pay dispersion on team performance. We collected a unique dataset from the Italian soccer league to study the effect of intra-firm pay dispersion on team performance, under different definitions of what constitutes a “team”. This peculiarity of our dataset can explain the conflicting evidence. Indeed, we also find positive, null, and negative effects of pay dispersion on team performance, using the *same data* but *different definitions of team*. Our results show that when the team is considered to consist of only the members who directly contribute to the outcome, high pay dispersion has a detrimental impact on team performance. Enlarging the definition of the team causes this effect to disappear or even change direction. Finally, we find that the detrimental effect of pay dispersion is due to worse individual performance, rather than a reduction of team cooperation.

## Introduction

Does pay dispersion have a positive or negative effect on work and organizational performance [1]–[2]? Pay dispersion is a property of a pay distribution, which is the “array of compensation levels paid for differences in work responsibilities, human capital, or individual performance within a single organization” [3]. The literature presents mixed evidence on the relation between the level of pay dispersion within an organization and work performance [4]–[5]. From one perspective (mainly social psychology), pay dispersion is believed to cause perceptions of inequity and relative deprivation that are detrimental to cooperation [6]. From another perspective (economics), pay dispersion can motivate employees located near the bottom of the pay-distribution scale to work harder for a future reward—a higher salary [7]–[8] —particularly when the pay dispersion is viewed as legitimate [9]. Pay dispersion may also be beneficial for attracting and keeping talent [10] or for avoiding the loss of workers who are crucial to the firm's output [11]. To add to the already blurry picture, some research finds no significant relation between pay dispersion and work performance [12]–[14].

In this study we focus on teams because teams are fundamental, and increasingly common, units of organization [15]–[18]. Teams are adopted because of their potential synergies. Thus, “team production may expand production possibilities by utilizing collaborative skills” [19]. Research suggests that firms increasingly organize around teams [20]–[21]. At the same time, firms also increasingly differentiate rewards [22], such that the firm-level dispersion of pay is widening. The two trends may be related, as smaller organizational units (i.e., teams) are associated with smaller costs of measuring and, therefore, rewarding input and output performance [23]. Although pay dispersion may exist between teams, pay may also be differentiated within teams [24], especially within top-management [25] and professional sports teams [26]. This raises the issue of the effects of within-team pay dispersion on team performance.

The empirical evidence concerning the relation between pay dispersion within teams and performance is mixed and inconclusive. Some studies support the idea that pay dispersion has a beneficial effect on team performance [27]–[29]; other studies show that pay dispersion has a detrimental effect [30]–[33] further studies find no significant effect [34]–[36].

In this study we show that the estimates of the effect of pay dispersion vary when using different definitions of what constitutes a team. Pfeffer and Langton [37] note that “one of the more useful avenues for research on pay systems may be precisely this task of determining not which pay scheme is best but, rather, under what conditions salary dispersion has positive effects and under what conditions it has negative effects.” We provide evidence that the effect of pay dispersion can be positive, null, or negative depending on the precision of the definition of team. Our dataset in fact allows us to measure pay dispersion by distinguishing between “active” and “passive” players. Both are part of the team, but only the former ones contribute to the team's performance.

Our dataset is drawn from two seasons of the men's major soccer league in Italy. Professional sports data represent a unique source of data for labor market research, and they are widely used because they provide detailed statistics about team performance, as well as the individual athletes' performances and salaries. Soccer is a particularly appropriate area of study for our research question for a number of reasons. First, it is a team sport where cooperation is crucial, although teams may also win (lose) because of extraordinarily good (bad) individual performance. Second, it is possible to identify each individual's participation (in terms of minutes played) and to obtain repeated measures of performance over time (multiple matches in one season). Third, these data are reliable, detailed, and reported with high precision. Our dataset contains information on the net salary of each team member, and statistics on each team, each team member, each head coach, and each match. Fourth, this sport is one of the best known and popular in the world, particularly in Italy, where it generates revenues of about 1,5 billion euros [38]. Given this popularity, players' salaries are highly publicized in the media. This means that each player is aware of the pay of his teammates, at least until the opening of a new session of the players' transfer market (each January).

Another important reason why we decided to use soccer data is that each team roster usually consists of around 25 to 30 athletes, but only 11 to 14 of them actually play a single match, with a moderate turnover from one match to another. Therefore, these data allow us to measure the effect of pay dispersion using various definitions of team and provide an explanation for why the previous literature has found mixed evidence. The existing literature cited above [39] looks at end-of-season data, comparing the wins-to-matches ratio with the pay dispersion of the entire team roster, paying no attention to individual contributions to team performance. To the best of our knowledge, our study is the first to compare the outcome of a single task (a match) with the pay dispersion of only those who contributed to the task. We believe our approach improves the precision of the comparison and can shed new light on our understanding on the effect of pay dispersion on work performance.

Our data show that the within-team variation of pay dispersion is related only to the number of injured and disqualified players, but not to the characteristics of the opponent team or whether the team plays at home or away. This suggests that pay dispersion is not chosen strategically by the coach and endogeneity does not seem to play a role in our dataset. As a further control, in our analysis we study the relation between team performance and pay dispersion, including in the specification several characteristics of the team, the head coach, the match and the opponent team. Repeating the analysis on a sub-sample of teams homogeneous in terms of pay size, age, and experience would even reinforce our results.

Our findings are clear-cut. Using the narrowest definition of a team, that is, considering only those who played the match and how long they played for, pay dispersion has an overall negative impact on team performance; this result is consistent with different robustness checks. However, that effect changes—and it may even become significantly positive—when we enlarge the definition of team to include the entire team roster. We interpret this result as the consequence of taking an approximation of the correct pay dispersion where a less precise definition can bias the estimates.

Different scenarios may explain the negative effect of pay dispersion on team performance.In particular, the effect may come about because high pay dispersion affect team performance through lack of cooperation among team members or it comes about through lack of individual effort. To understand which explanation is supported by data, we collected all (subjective) individual performance assessments for each match, for each team, and for each player reported by the three most popular Italian sports newspapers. Our results show that higher pay dispersion has a detrimental impact on individual performances, but has no significant effect on cooperation. There is, however, a third possibility that our data unfortunately does not allows us to satisfactorily address and resolve. Specifically, there is the possibility that pay dispersion reflects a dispersion of the skills, abilities and talents of players, and that the association between pay dispersion and decreased team performance comes about because a high disparity of skills, etc. makes the team play less well together. For example, more homogenous players coordinate efforts better.

Finally, our analysis controls for pay size and we use indicators of pay dispersion that are dimensionless. For this reason, our results can be extended to other work contexts, beyond the peculiar work environment of professional sports. Our findings may be able to help managers determine which type of pay distribution will be more effective within a firm and make the right decisions about which employees to hire. For example, should a firm hire one expensive superstar employee and two inexpensive employees, or three medium-priced employees? We provide numerical examples showing that managers should carefully take into account the hidden cost of hiring a superstar and its effect on team performance, while keeping constant the overall team quality.

## Data and Estimation Methodology

### Empirical Setting and Data

Our data cover the two seasons 2009–2010 and 2010–2011 of the men's major soccer league in Italy (“Serie A”). Every season 20 teams participate in the league, and each team plays against each other team twice (one time at the home stadium and one time away) for a total of 38 matches. After a match three points are assigned for a win, one point for a draw, and no points for a defeat. The ultimate goal of each team is to earn points and be classified as high as possible in the league's ranking in order to win it or at least be in the top six positions and in this way gain access to the European cups. Teams also want to avoid being placed in any of the bottom three positions, which would relegate them to the second division. In fact, at the end of each season, the three teams ranked last are replaced by the three teams ranked first in the second division.

Our dataset contains information on the outcome of each match (win, draw, or defeat), on who played every single match and for how many minutes, and his annual net pay, as well as other statistics on each player, on each team, on each head coach, and on each match. We collected this unique dataset by merging data from the three most popular Italian sports newspapers (*La Gazzetta dello Sport*, *Corriere dello Sport*, and *Tutto Sport*), and (for players' statistics) from the website www.tuttocalciatori.net.

In any season, each team consists of about 25 to 30 athletes (henceforth, the team roster) specializing in different roles (goalkeeper, defender, midfielder, forward). However, only 18 members are summoned for each match: 11 (starter players) start the game and the other 7 (substitutes) sit on the bench and can enter the match at any time after the beginning, replacing one of the starter players (who can no longer take further part in the match). During a match a maximum of three substitutions is allowed. Common reasons for substitutions include injury, tiredness, ineffectiveness, or a tactical switch. In the 2009–2010 season 462 players and in the 2010–2011 season 463 players played for at least one minute during our observation period. In most cases those who played in one season also played in the other one; however, from our perspective they are completely different players because they may earn different salaries in the two seasons. For this reason and for sake of simplicity and with a little abuse of terminology, we say that 925 team members have played overall. A similar argument can be made for teams: because those teams present in both seasons may have very different lists of team members, we treat them as different teams, so that our sample includes 40 teams. We know the salaries of only 874 of the 925 players (94.49%), while we impute the pay of the remaining 51. This imputation has a negligible impact on our statistics, because players for whom we needed to impute salaries have a marginal role in the team (on average they have played about 1% of the available time). Repeating our benchmark analysis without imputations (results available upon request) confirms our findings.

Our dataset includes the matches played between August 23, 2009, and December 20, 2009 (2009–2010 season), and between August 29, 2010, and December 19, 2010 (2010–2011 season), for a total of 666 observations. To be conservative and have a clean dataset, we decided to use only the matches played before the opening of the January players' transfer market, during which every team is allowed to trade players with other teams. We then ignored the remaining matches, for which we cannot be sure about the exact salaries of players transferred, especially the ones coming from foreign leagues. On average these players account for around 12% of the team members after the January market, and they usually take a relevant role in the team—playing most of the remaining matches. If we included these data, any guesses about the missing salaries would likely bias our estimates. However, there is relatively high correlation (0.589) between the number of points earned in the first 17 matches and the number of points earned in the remaining matches.

### Variables and Estimation Method

Our unit of analysis is the team playing a match in a given season; in total we then have 40 teams, 20 for each season. Recall that the team may win, draw, or lose a match, earning respectively three, one, or no points. Our dependent variable, measuring team performance, is a dummy equal to 1 if the team wins the match (which happens in 37.24% of the cases); it is equal to 0 if the team draws or loses the match. We group draws and defeats together because the ultimate goal for a team is to win a match. In a robustness check, we repeat the analysis treating first both wins and draws as a positive outcome, and then each outcome separately. Our main results were confirmed (Supplementary material available upon request).

We perform a probit regression with panel-robust standard errors (clustered for each team in each season); this way we allow for possible correlation across observations referring to different matches of the same team. We opted for this model because our data show no evidence of team-specific panel effects (see the discussion at the end of Section 3.1); use of this model allows us to obtain more efficient estimates.

Our purpose is to obtain measures of pay dispersion, as well as other indicators, that are specific for each match of each team. For this purpose, the term *active team members* (ATMs) for a team in a given match refers to all team members who actually played at least one minute of the match. For such a match we then neglect all of the remaining members who did not contribute to the result of the match. As a consequence, the set of active team members for a team varies match by match.

The benchmark specification includes different variables that for clarity we group into six categories: *pay, team, coach, match*, *opponent*, and *time*. Our focus is on the first group of pay variables; the remaining ones serve as control variables. In the analysis, all of the variables concerning team composition are based solely on the ATMs, and the contribution of each member is weighted by the amount of time he actually played in the match. The variables in the *pay* and *team* categories thus refer to the ATMs of the team, whereas the variables in the *opponent* category refer to the ATMs of the opponent team. This means that *pay*, *team*, and *opponent* statistics differ match by match and that members who had no active role in the match are ignored. In what follows we discuss the variables used in the analysis.

#### Pay variables

We consider the logarithm of the average pay, and the logarithm of a dimensionless measure of pay dispersion. In all the cases we refer to annual salaries in thousands of euros net of taxes. Let us define 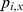 as the pay of player 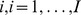 in team 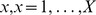, where 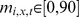 represents the minutes of the match actually played by the same player in match 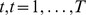. We treat 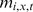 as a weight to compute the average pay for team *x* in match *t*:
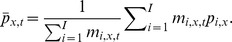



As a pay dispersion measure, we take the Theil index. This indicator belongs to the class of entropy indexes and is frequently used to measure economic inequality. The index is defined as the mean of the products between individual pay relative to average pay, and its logarithm is as follows:



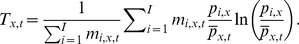



The index is equal to 0 for the case of no pay dispersion (i.e., all salaries are identical); a higher index denotes higher pay dispersion. Notice that the indicator is dimensionless, which means that what matters to us is only the individual pay *relative to the average pay*; this allows us to compare pay distributions across teams and matches, disregarding the average pay level, which varies markedly (from 213,808 euros to 4,356,061 euros). As a robustness check, we repeated the analysis using the popular Gini index rather than the Theil index. In this case our main findings were qualitatively confirmed, and quantitatively even emphasized (Supplementary material available upon request).

#### Team variables

We use weighted average values in a given match for players' ages, the fraction of new players on the team, and the number of years (even if not consecutive) on the team and in the Italian first division; the last two variables serve to proxy players' experience.

#### Coach variables

For the coach we use the same set of information as for the team, that is: coach age, a dummy variable equal to 1 if he is in his first season with the team, and the number of years (even if not consecutive) on the team and in the Italian first division. Head coaches in soccer are often fired from one season to another, and even during the same season. We then also include in the analysis a dummy variable equal to one if the head coach has been replaced during the season.

#### Match variables

We use a dummy variable equal to 1 if the team plays a domestic (home) match. In addition, soccer players may be sanctioned with a yellow or red card for a specific misconduct; multiple yellow cards or one red card produce an automatic disqualification for at least one following match. We therefore use the number of disqualified players as well as the number of injured players. These variables are added because injuries and disqualifications may prevent a coach from using his preferred players during a match. However, we expect disqualifications to have a stronger effect because they usually involve team members who play more frequently.

#### Opponent variables

We consider the same variables as in the *pay* and *team* groups, but we base them on the ATMs of the opponent team. The purpose is to in this way capture the characteristics (in particular the strength) of the opposing team. An alternative would be to add as many dummy variables as the teams (40). Doing so, our main results would be confirmed. The shortcoming of such an approach, however, is the potential inefficiency of estimating many coefficients in a probit regression model; for this reason we prefer our benchmark specification.

#### Time variables

We use a dummy variable equal to 1 if the match was played during the 2010–2011 season, and dummy variables for the month if the match was played from August to December.

### Summary Statistics

Table 1 lists all variables used in the analysis and reports some summary statistics. All statistics are calculated for the ATMs in the 666 observations of our sample. The purpose of using all of these variables is to control for the physical, social, and other characteristics of the team members, the coach, and the match.

**Table 1:** Summary statistics (666 observations on 40 teams).

Variable	Median	Mean	Std. dev.	Minimum	Maximum
**Pay**					
Average pay (thousands of euros)	583.788	1039.801	998.699	213.808	4356.061
Theil index	0.093	0.114	0.077	0.006	0.497
**Team**					
Fraction of new players in the team	0.255	0.263	0.156	0	0.701
Years in the team	2.202	2.341	1.039	0.483	5.985
Years in first division	4.635	4.858	1.510	1.597	9.645
Average age	27.465	27.488	1.327	24.298	30.889
**Coach**					
New to the team	1	0.571	0.495	0	1
Replaced during season	0	0.144	0.351	0	1
Years in the team	0	0.685	0.982	0	4
Years in first division	3	4.372	3.686	0	14
**Age**	48	49.414	6.882	38	65
Match					
Injured players	3	3.081	1.798	0	11
Disqualified players	0	0.431	0.662	0	4
Home play	0.5	0.5	0.500	0	1

*Note: For the “opponent” variables we consider the same variables as in the pay and team categories, but we base them on the ATM of the opposing team. We do not report summary statistics because they coincide with those in the pay and team categories*.

From the table we learn that, for the median observation, the ATMs' average pay is 584 thousand euros, the Theil index is 0.093 (ranging from 0.006 to 0.497), on average the ATMs are around 27 years old, they already have accumulated two years of experience in the team and five years in the first division, and around 26% of them are in their first year with the team. In addition, 57% of the coaches are new to the team, 14% of them started managing the team after the beginning of the season, and they have little experience with the team and the first division. Finally, disqualifications and especially injuries are frequent, and sometimes they may force the coach to reshape the starting team formation (in fact, we observe a maximum of 4 disqualified players and 11 injured players). In our analysis we account for this when measuring the effect of pay dispersion; further statistics on pay dispersion are shown in the supplementary material (available upon request).

Table 2 lists the teams in our dataset (20 for each season) and some average statistics (age, experience, fraction of new players) for their ATMs in each match. Teams are listed according to the ranking at the end of each season, where the first team listed is the winner of the championship and the last three teams are eventually relegated to the second division.

**Table 2:** Team statistics.

a) 2009–2010 Season					
Team	Fraction new to the team	Years on the team	Years in first division	Age	Fraction of players employed
FC Internazionale Milano	0.314	3.553	4.961	29.320	0.733
AS Roma	0.093	3.755	6.278	28.378	0.871
AC Milan	0.128	4.538	8.063	29.619	0.786
UC Sampdoria	0.265	1.862	5.751	26.639	0.778
US Città di Palermo	0.188	1.650	4.085	25.886	0.852
SSC Napoli	0.289	1.465	4.743	26.897	0.808
Juventus FC	0.239	3.056	5.838	28.460	0.929
Parma FC	0.535	0.847	6.160	27.604	0.808
Genoa CFC	0.359	1.527	4.421	27.760	0.786
AS Bari	0.435	1.507	2.836	26.267	0.774
ACF Fiorentina	0.110	2.695	7.519	27.818	0.750
SS Lazio	0.042	2.582	5.689	27.455	0.806
Catania Calcio	0.282	1.650	2.333	26.120	0.893
Cagliari Calcio	0.134	3.406	4.695	26.910	0.720
Udinese Calcio	0.131	2.370	4.312	25.502	0.733
AC Chievo Verona	0.199	2.647	4.622	29.198	0.852
Bologna FC	0.439	0.810	5.098	29.076	0.815
Atalanta Calcio	0.253	2.513	4.024	26.915	0.923
AS Siena	0.302	1.692	4.203	26.550	0.885
AS Livorno	0.310	1.552	3.689	27.437	0.800
**AVERAGE**	**0.252**	**2.280**	**4.962**	**27.491**	**0.834**

b) 2010–2011 Season					
Team	Fraction new to the team	Years on the team	Years in first division	Age	Fraction of players employed
AC Milan	0.228	4.749	7.927	29.480	0.828
FC Internazionale Milano	0.108	3.786	5.430	29.308	0.862
SSC Napoli	0.166	1.766	5.393	27.573	0.917
Udinese Calcio	0.166	3.117	4.723	25.740	0.909
SS Lazio	0.196	2.130	4.479	27.918	0.846
AS Roma	0.188	4.001	7.135	29.506	0.963
Juventus FC	0.542	2.066	5.297	27.173	0.926
US Città di Palermo	0.308	1.555	3.299	24.939	0.692
ACF Fiorentina	0.124	2.960	7.440	27.659	0.929
Genoa CFC	0.499	1.550	4.310	27.718	0.846
AC Chievo Verona	0.434	2.065	3.594	27.916	0.880
Parma FC	0.311	1.358	5.315	27.914	0.769
Cagliari Calcio	0.077	3.281	4.391	26.090	0.760
Catania Calcio	0.083	2.255	2.887	27.144	0.889
Bologna FC	0.399	0.902	3.363	26.605	0.923
AC Cesena	0.494	1.703	4.168	28.302	0.852
US Lecce	0.401	2.446	2.529	27.306	0.926
UC Sampdoria	0.124	2.258	5.975	26.046	0.929
Brescia Calcio	0.383	2.446	4.015	28.541	0.960
AS Bari	0.236	2.322	3.679	27.012	0.929
**AVERAGE**	**0.274**	**2.401**	**4.755**	**27.484**	**0.873**

*Note: Teams are listed according to their position at the end of the season; teams promoted from second division are highlighted. Averages for each team are based on the ATM of all of the matches (either 16 or 17) played by the team in a given season. Fraction of players employed: number of players employed at least for one minute over total number of players*.

First of all, we notice that the 17 teams enrolled in both seasons show marked differences over the two years. From the table we also observe wide heterogeneity across teams within the same season, with no clear pattern going from bottom to top teams. The last column of Table 2 shows the fraction of players that in our sample played at least for one minute. This fraction is between 0.69 and 0.96; note that it is always below 1. This indicates that some team members never play; usually those excluded are injured and homegrown players. Ignoring this, and treating all team members equally, the analysis on the effect of pay dispersion may generate different results, as we later clarify.

**Figure 1:**
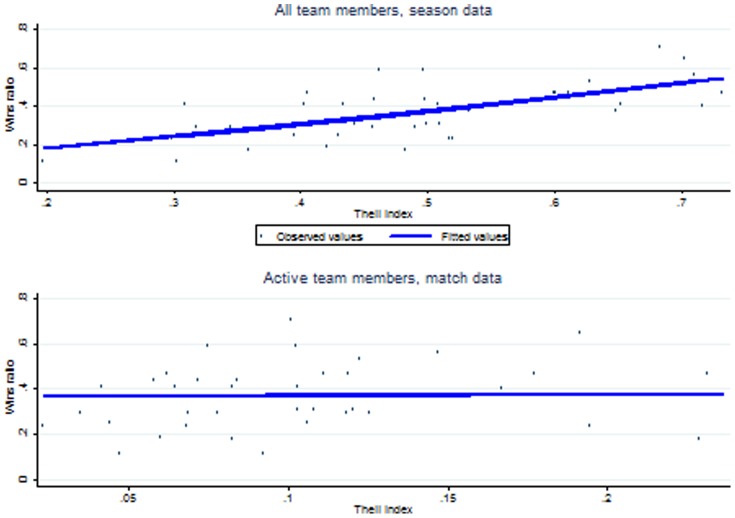
Team performance and pay dispersion (40 team observations).

To stress this point, Figure 1 plots for each team the fraction of wins over the Theil index, using two different methods. In the top panel, the pay dispersion index is based on the whole team roster, disregarding players' involvement in the matches; this is the standard approach adopted in the literature. In the bottom panel, the index is the average over the matches, where for each match pay dispersion is based on the ATM; this approach is closer to the one followed in this paper. First of all, we notice that the index calculated in the top panel uses values that are on a higher scale than those of the index in the bottom panel; the reason is that this measure is inflated by the low pay of those members (usually the homegrown ones) who, although formal members of the team, do not contribute to the team's performance.

The figure also shows a line indicating the predicted winning probability for a given level of the Theil index. The prediction is obtained from a simple probit regression over 666 observations, where the dependent variable is equal to 1 if the team wins the match, and 0 otherwise; the specification includes just the constant and the Theil index, based on either the whole team roster (top panel) or the ATM of each match (bottom panel). Comparing the two panels, we see that pay dispersion positively affects team performance when considering the whole team roster (top panel), whereas it has no impact when considering the ATMs (bottom panel). This suggests that results may change depending on how pay dispersion is measured. This finding warns us that findings may change depending on our definition of what constitutes a “team.”

We conclude this section with an exploratory analysis of the effect of pay dispersion on performance, which is our ultimate goal. Overall in the data, pay dispersion shows no significant difference (*t* test: 0.37; *p* value: 0.712) when the match is won (average: 0.116) or when the match is drawn/lost (average: 0.114). Pay dispersion is not even affected by the team performance of the previous match (*t* test: 0.607; *p* value: 0.544; average after a match won: 0.117; average after a match drawn/lost: 0.113). This suggests that the coach does not adjust it to keep the team compact in case of performance problems.

Table 3 then shows, separately for each team, the average pay, the average Theil index, and the wins ratio. Teams are listed as in Table 2, following their ranking at the end of the season. The first thing to note brings to mind the famous slogan “*The more you spend, the more you get*.” Indeed, teams that spend more (i.e., with a higher average pay) rank higher at the end of the season. In fact, our data exhibit a large Spearman's rank correlation (0.701) between average team pay and the wins ratio in the season. The data thus suggest that better players are also better paid, and for this reason we can interpret the average pay of a team as a proxy for the average skill in the team. In contrast, pay dispersion is much less highly correlated with the wins ratio (the rank correlation is 0.241), although the sign of this correlation is still positive.

**Table 3:** Pay and team performance.

a) 2009–2010 Season						
Team	Average pay	Theil index	Wins ratio: average by matches			(2)–(1)>0
			All	Low disp.	High disp.	
				(1)	(2)	
FC Internazionale Milano	4115.021	0.101	0.706	0.875	0.556	NO
AS Roma	1718.652	0.232	0.471	0.375	0.556	YES
AC Milan	3250.733	0.147	0.563	0.750	0.375	NO
UC Sampdoria	724.647	0.393	0.412	0.750	0.111	NO
US Città di Palermo	658.497	0.083	0.412	0.500	0.333	NO
SSC Napoli	842.041	0.103	0.412	0.500	0.333	NO
Juventus FC	2673.181	0.123	0.529	0.625	0.444	NO
Parma FC	536.108	0.062	0.471	0.500	0.444	NO
Genoa CFC	817.235	0.058	0.438	0.500	0.375	NO
AS Bari	435.087	0.126	0.375	0.125	0.625	YES
ACF Fiorentina	1177.068	0.072	0.438	0.250	0.625	YES
SS Lazio	729.187	0.229	0.176	0.125	0.222	YES
Catania Calcio	413.871	0.048	0.118	0.125	0.111	NO
Cagliari Calcio	367.552	0.084	0.438	0.625	0.250	NO
Udinese Calcio	464.575	0.103	0.313	0.250	0.375	YES
AC Chievo Verona	380.442	0.042	0.412	0.500	0.333	NO
Bologna FC	523.939	0.106	0.250	0.375	0.125	NO
Atalanta Calcio	334.134	0.060	0.188	0.125	0.250	YES
AS Siena	436.847	0.083	0.176	0.250	0.111	NO
AS Livorno	358.900	0.125	0.294	0.500	0.111	NO

b) 2010–2011 Season						
Team	Average pay	Theil index	Wins ratio: average by matches			(2)–(1)>0
			All	Low disp.	High disp.	
				(1)	(2)	
AC Milan	3590.499	0.191	0.647	0.750	0.556	NO
FC Internazionale Milano	3250.635	0.167	0.400	0.429	0.375	NO
SSC Napoli	918.736	0.102	0.588	0.625	0.556	NO
Udinese Calcio	568.033	0.065	0.412	0.500	0.333	NO
SS Lazio	1099.115	0.075	0.588	0.750	0.444	NO
AS Roma	2049.492	0.177	0.471	0.625	0.333	NO
Juventus FC	2034.127	0.119	0.471	0.500	0.444	NO
US Città di Palermo	576.675	0.111	0.471	0.500	0.444	NO
ACF Fiorentina	1024.088	0.108	0.313	0.250	0.375	YES
Genoa CFC	1128.980	0.236	0.375	0.500	0.250	NO
AC Chievo Verona	316.556	0.035	0.294	0.375	0.222	NO
Parma FC	559.824	0.068	0.235	0.250	0.222	NO
Cagliari Calcio	403.838	0.078	0.294	0.250	0.333	YES
Catania Calcio	447.094	0.069	0.294	0.250	0.333	YES
Bologna FC	555.712	0.119	0.294	0.125	0.444	YES
AC Cesena	223.423	0.044	0.250	0.250	0.250	=
US Lecce	300.007	0.023	0.235	0.375	0.111	NO
UC Sampdoria	897.708	0.121	0.313	0.250	0.375	YES
Brescia Calcio	359.365	0.195	0.235	0.250	0.222	NO
AS Bari	482.681	0.093	0.118	0.250	0.000	NO

*Note: See note to* *Table 2* *. Average pay is in thousand euros. For each team we split matches in two groups based on whether the Theil index was below (low) or not below (high) the median value for the team*.

A problem with this analysis is that it ignores the specific characteristics of each team. For this reason, we now compare, separately for each team, the wins ratio obtained in two groups of matches, where the Theil index is either below or above the median for the team. The last column of Table 3 shows that the wins ratio is higher in the matches with high pay dispersion in only 11 cases out of 40.

We have then found that, looking at the same data, one can interpret the relationship between team performance and pay dispersion as positive (considering all the team members: Figure 1, top panel), null (considering the ATMs: Figure 1, bottom panel), or negative (considering the ATMs separately for each team: Table 3). Our empirical exercise in the next section further analyzes the relationship considering the ATMs, each match separately, and controlling for the most relevant characteristics of the team, the coach, the match, and the opponent.

## Pay Dispersion and Team Performance

In this section we summarize our main findings regarding the effect of pay dispersion on team performance. We then discuss some robustness checks around the definition of team members, and we report the results of a further analysis connecting pay dispersion with individual performance. Our benchmark estimates are shown in Table 4.

**Table 4:** Team performance and pay dispersion (average marginal effects).

		(1)	(2)	(3)	(4)
Members:		ATM	Unweighted	Potential	Roster
Pay:	Log(average pay)	0.146***	0.142***	0.147***	0.076**
		(0.027)	(0.028)	(0.032)	(0.039)
	Log(pay dispersion index)	−0.061**	−0.058*	−0.046	0.167**
		(0.030)	(0.032)	(0.031)	(0.082)
Team:	Fraction of new players on the team	0.095	0.040	0.059	0.044
		(0.143)	(0.148)	(0.144)	(0.141)
	Years on the team	0.015	0.011	0.020	0.006
		(0.026)	(0.029)	(0.030)	(0.037)
	Years in first division	0.002	−0.004	−0.006	0.006
		(0.016)	(0.018)	(0.018)	(0.022)
	Age	0.001	0.014	0.005*	0.009
		(0.017)	(0.016)	(0.002)	(0.017)
Coach:	New to the team	−0.091*	−0.086*	−0.089*	−0.116**
		(0.050)	(0.050)	(0.047)	(0.046)
	Replaced during the season	0.119**	0.115**	0.120**	0.108**
		(0.051)	(0.054)	(0.056)	(0.048)
	Years on the team	−0.034**	−0.033**	−0.032**	−0.033**
		(0.015)	(0.016)	(0.015)	(0.016)
	Years in first division	0.010*	0.011**	0.011**	0.009*
		(0.006)	(0.006)	(0.005)	(0.005)
	Age	0.000	−0.000	0.000	−0.000
		(0.004)	(0.004)	(0.004)	(0.003)
Match:	Injured players	−0.006	−0.005	−0.004	−0.007
		(0.010)	(0.010)	(0.010)	(0.010)
	Disqualified players	0.040*	0.040*	0.040*	0.024
		(0.023)	(0.023)	(0.023)	(0.022)
	Home play	0.253***	0.250***	0.248***	0.256***
		(0.034)	(0.035)	(0.033)	(0.034)
Opponent:	Log(average pay)	−0.169***	−0.160***	−0.183***	−0.125***
		(0.029)	(0.032)	(0.035)	(0.037)
	Log(pay dispersion index)	0.030	0.018	0.015	−0.083
		(0.025)	(0.026)	(0.033)	(0.095)
	Fraction of new players on the team	0.139	0.206*	0.088	0.183
		(0.105)	(0.119)	(0.120)	(0.153)
	Years on the team	0.044*	0.048*	0.045	0.084**
		(0.022)	(0.027)	(0.029)	(0.041)
	Years in first division	0.001	0.004	0.012	−0.014
		(0.015)	(0.016)	(0.017)	(0.025)
	Age	0.004	−0.007	−0.003**	−0.019
		(0.015)	(0.017)	(0.001)	(0.020)
*+ time dummy variables on month and year of the match*					
	Log-likelihood	−371.461	−371.522	−370.738	−371.522
	McFadden R^2^	0.155	0.155	0.157	0.155
	Count R^2^	0.689	0.688	0.700	0.688
	Rho coefficient	0.000			
	LR test rho = 0	0.000			
		[0.496]			

*Note: 666 observations on 40 teams (on average, 16.6 matches per team). The dependent variable is a dummy  = 1 in case of win. Pay and team statistics are based on ATM players (column 1); ATM players, not weighted by the amount of time they actually played (column 2); all potential players (starter players and substitute players; column 3); whole team roster (column 4). Team-clustered standard errors are given in parentheses; p values in brackets*.

*p<0.1;

**p<0.05;

***p<0.01.

### Benchmark Analysis

**Figure 2:**
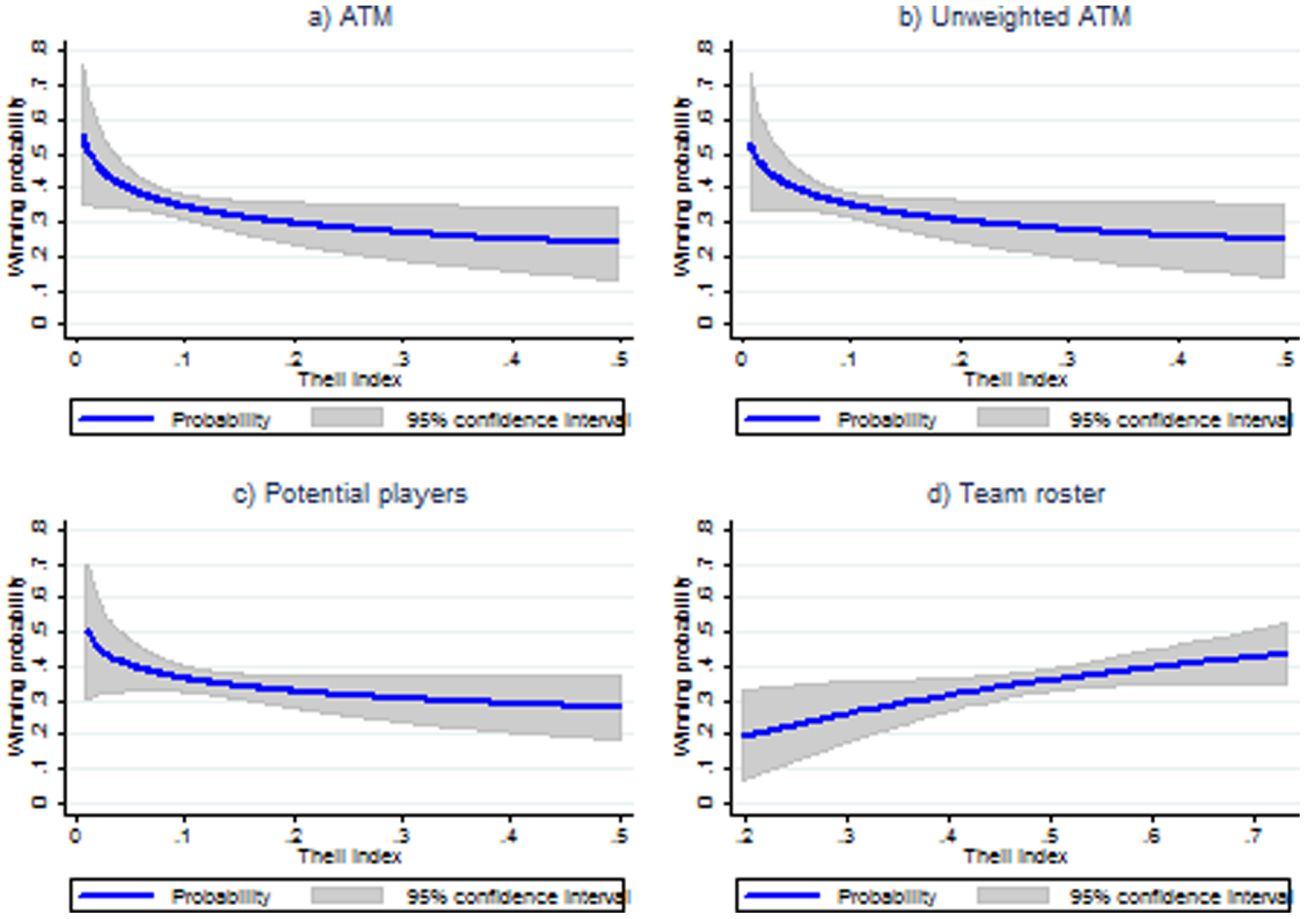
Predicted winning probability by pay dispersion. Note: Predictions are based on the average explanatory variables and the parameter estimates from Table 4.

The first column of Table 4 reports the average marginal effects from our benchmark probit regression analysis. The column shows that pay dispersion has a *negative impact* on team performance: doubling pay dispersion, the probability of winning a match would reduce on average by 0.06. Panel (a) of Figure 2 plots the predicted winning probability, conditional on pay dispersion and the other explanatory variables (fixed to their average), computed using this probit regression. It shows that probability falls, from 0.56 when there is no pay dispersion, to 0.24 when the Theil index is *T* = 0.50.

An example will help the reader understand this figure. Suppose a team manager has to buy 11 new players who are expected to play all the next matches fully and on a regular basis. Your budget is limited, and you have to decide whether to buy (at the same total expenditure) either 1 top player and 10 average players, or instead 11 players with above-average skill. We assume their pays reflect their skill. Further, let us say that the average pay is 600 thousand euros (the actual pay size in our sample; however, this is irrelevant for the pay dispersion index) and that the manager can choose to pay all 11 players the same amount (600 thousand euros) or 1 top player much more (1.5 million euros as opposed to 510 thousand euros for the other ones). In the latter case the top player will earn 2.5 times the average pay, while each other player will earn 0.85 times the average pay; this pay distribution roughly corresponds to the median distribution in the sample. The resulting Theil index is *T* = 0.083, whereas it is *T* = 0 if all 11 players earn 600 thousand euros each. Hence, higher pay dispersion denotes higher variability of players' skills. Our estimates suggest that, everything else being equal, the differentiated pay distribution will make the probability of winning a match fall on average by 20%, from 0.56 to 0.36.

In our regression we also find significant evidence of a positive effect of average pay (doubling it would increase the probability of winning a match by around 0.15), replacing a coach during the season (the probability then increases by 0.12), and playing at home (0.25). In addition, we find significantly negative effects for the coach's experience with the team (one more year reduces the probability of winning a match by 0.03) and the opponent's average pay (doubling it would reduce the probability by 0.17). These results are not surprising: on average, the pay can be seen as a proxy for a player's skill (above we made an argument about this); replacement of a coach during the season may have a large psychological impact on the players; a team playing in its home stadium may benefit from the support of its fans; the longer a coach is on the team, the lower is the strength of his effort and the psychological impact on the players; and the opponent's average pay can also be seen as a proxy for its skill, which then lowers the winning probability of the team. No other explanatory variables—noticeably, those on the team characteristics and on the opponent's pay dispersion—are significantly different from 0, at least at a 5% significance level.

The “rho” coefficient, shown in the bottom part of Table 4, is the proportion of the total variance contributed by the team-level variance. This is statistically equal to 0, indicating that we can disregard the panel dimension of our data, and run our analysis with a probit regression on the pooled dataset. In what follows we then perform pooled probit regressions with team-clustered standard errors, because this approach is more efficient than using panel regression methods (fewer parameters have to be estimated).

In an additional analysis (Supplementary material, Section B.1; available upon request), we discuss the link between pay dispersion and the main characteristics of the match and the team opponent, showing positive correlation with the number of injured and disqualified players, but no correlation with the characteristics of the opponent team or whether the team plays at home or away. This suggests that match-by-match variations in pay dispersion are not driven by strategic reasons. The section then replicates our analysis on a sub-sample of teams homogeneous in terms of pay size, age and experience. In this case, the effect of pay dispersion on team performance would remain negative, but larger than in the benchmark analysis (−0.16 rather than −0.06). Section B.2 repeats the benchmark analysis, also treating draws as a positive outcome, and confirms our benchmark results. Moreover, Section B.3 reports the results of some robustness checks on the specification, where we substitute the Theil index with the Gini index (which actually shows a stronger significant effect: −0.13 rather than −0.06), or where we add an indicator of the symmetry of the pay distribution (eventually not significant), a quadratic polynomial on pay dispersion, or the interaction between the index and a dummy variable equal to 1 if the team played a match in December. In the latter two cases, the purpose is to understand whether the effect of pay dispersion is non-monotonic or if it changes as team members get to know each other better. In neither case are the added variables significantly different from 0. In addition Section 3.3 discusses, among other things, the relationship between team performance and different technologies of production.

### Team Members

We repeat the analysis with the same regression specification as in the benchmark case, but this time we consider different definitions of team members. As we have already seen, the definition affects the computation of the variables on pay, team, and opponent statistics that are all match specific. The effect of pay dispersion on team performance may then change with the definition of group. The average marginal effects from the analysis are shown in columns (2), (3), and (4) of Table 4; the latest column is based on the broadest definition of team members.

#### Unweighted ATM

We first consider the ATM, as in the benchmark, but disregarding the amount of time they actually played. For instance, if the match started with 11 players and then 3 substitutes also took part in the match, we derive our pay and players statistics from the characteristics of 14 team members, without weights.

Our results are reported in column (2) of Table 4, and they are close to the benchmark case of column (1). In particular, pay dispersion is still associated with a negative marginal effect of −0.06, although the effect is now significant at only 10%. This suggests that ignoring the amount of time spent in the field may create noise in the estimates.

#### Potential players

We then consider as team members all 18 athletes who were potentially able to play in the match because they were either starter players or substitute players. In this manner we exclude injured players, disqualified players, or players who are out of the match as a result of a decision made by the coach. All members are given the same weight, disregarding the number of minutes they actually played in the match. This definition of team members is less precise than our benchmark definition of ATMs, because at least four of these members in each match make no contribution to team performance, but they still affect the pay, team, and opponent statistics.

Our results are shown in column (3) of Table 4. Most variables show effects that are in line with the benchmark results; however, the pay dispersion index is now associated with a coefficient insignificantly different from zero.

#### Entire team roster

We conclude the analysis by considering as team members all athletes enrolled on the team, that is, the entire team roster, thus including injured, disqualified, and homegrown players. Hence, we consider the same team composition in each match, disregarding who actually played. This implies that, in our regression equation, the variables on pay and team statistics are constant for a given team (they are then fixed “team effects”), and the variables on opponent statistics are constant for a given opponent team. Such an approach is similar to that of some previous works in the literature, because it does not pay attention to whether and how much each team member contributed to team performance.

Our results are shown in column (4) of Table 4, and they are largely different from our benchmark analysis of column (1): we find a smaller effect of the team average pay (0.08 instead of 0.15), while the effect of pay dispersion is now even positive: according to these estimates, doubling pay dispersion would increase the probability of winning by 0.17. In contrast, the remaining variables, which have not changed relative to the benchmark case (they do not depend on the definition of team members), provide parameter estimates comparable with those of the benchmark case.

The results in Table 4 thus inform that, when broadening the definition of team (i.e., when going from column 1 to column 4), conclusions about the effects of pay dispersion change enormously: at a 5% level we may indeed find either a negative effect (column 1), a null effect (columns 2 and 3), or a positive one (column 4). Figure 2 plots the predicted winning probability, conditional on pay dispersion and the other explanatory variables (fixed to their average), computed separately from each of the four probit regressions in Table 4. From the figure it is clear that the direction of the effect goes from negative to positive as we use less information on the group definition, from panel (b) (where we ignore the amount of time actually played) to panel (c) (where we consider all starter and substitute players), and on to panel (d) (where we include the whole team roster).

This result is essentially a warning that the measurement of an effect can be biased if we do not consider a precise definition of what constitutes a “team.” Notice in particular that we find a positive effect when we look at the most general definition (whole team roster). Those who play little or not at all usually earn less than those who play regularly. See supplementary material (available upon request), Section A.4, for details.

As a result, pay dispersion increases if we use a definition of team that incorporates them; in particular, considering the entire team roster, the index is on average 0.493, as opposed to 0.114 if we consider just the ATMs. Pay dispersion increases significantly more in the top 10 teams at the end of December of each season: the average difference between the pay dispersion index computed from the team roster and from the ATMs is on average 0.437 among the top teams, as opposed to 0.321 among the other teams (*t* test: 16.512; *p* value: 0.000). The pay dispersion index then captures part of the effect of the team skill; indeed, the correlation between average pay and pay dispersion is 0.722 using the whole team roster, whereas it is only 0.253 using the ATM. This correlation may explain why in column (4) of Table 4 the effect of pay dispersion is positive, and the effect of average pay is about half the effect found in the other three columns.

This suggests that our benchmark conclusions are not driven by a dataset with different features than others. Actually, our conclusions depend on the way we look at the data, and in particular on what we mean by “team members.” This may explain why in the literature we observe different results, and it shows the importance of the precision of the definition of team to evaluate the effect of pay dispersion.

### Individual Performance

So far the analysis has focused on objective indicators of team performance. Team performance, however, derives from individual performance and cooperation among team members. It is then possible that we observe poor team performance because there is *poor individual performance* or because there is *little cooperation*. For instance, in soccer, we can observe a poor team performance when each player tries to score without passing the ball to other players (lack of cooperation) or when each player prefers not to take the initiative but instead passes the ball to other players, thereby delegating to them the responsibility to score (the lack of effort). One may thus wonder what determines the detrimental effect of pay dispersion on team performance. Does pay dispersion work as a disincentive to individual effort? Alternatively, does pay dispersion merely decrease cooperation between players, leaving individual performance unchanged? These are the issues we want to address in this section.

Our data suggest that teams that win more often make significantly more passes during the match: the 20 teams winning more frequently on average make 410.47 passes, significantly more than the other teams making on average 383.52 passes (*t* test: 1.876; *p* value: 0.034). In Section B.2 of the supplementary material (available upon request), we report the output of a within-group panel regression analysis of the number of passes over the same specification as in the benchmark. We find no significant effect of pay dispersion. To the extent that the number of passes can be seen as a valid measure of team cooperation, the finding may be interpreted as an indication that team cooperation is not affected by pay dispersion. If our argument is correct, team performance is then affected solely by individual performance.

Obtaining an objective and thorough measurement of individual performance is impossible in our environment, because soccer is a team sport where few individual statistics are recorded compared to other sports such as baseball. (See, e.g., Scully (1974) for an analysis of the connection between individual performance and individual pay [40].) In addition, those few existing individual statistics record rare events (e.g., goals, assists, yellow cards) and are highly role specific (e.g., a forward player is more likely to score a goal than any other player). It would be difficult to use these statistics as measures of individual performance.

In Italy, however, it is quite common for journalists, when writing a newspaper report about a match, to assign a “mark” to each single player's performance. The mark is a number based on a scale from 0 to 10; a mark of 6 denotes fair performance and higher marks indicate good or excellent performance. This mark represents a *subjective individual performance assessment* (SIPA), because it is based only on the arbitrary opinion and taste of the journalist who attended the match. Still, it is a rough indicator of the individual performance of each team member and can be used to look at the effect of pay dispersion on individual team members. In this regard we collected all of the SIPAs for the players involved in the 333 matches considered in the main analysis, using the three major sport newspapers in Italy: *La Gazzetta dello Sport*, *Corriere dello Sport*, and *Tutto Sport*. To make SIPAs less heavily affected by the personal opinion of the journalists, we took an average SIPA from the three newspapers (the SIPAs from the three sources show a correlation of around 0.7). Overall we have 8,226 observations on 876 players (434 in the 2009–2010 season and 442 in the 2010–2011 season), who then played an average of 9.39 matches each. The number of players considered is smaller than the number of players who played at least for one minute, 925, because marks are given only to those who play a significant portion of the match. The decision on what is a “significant portion of the match” is subjective, and different journalists may have different opinions. In a separate analysis in the supplementary material (available upon request), Section B.4, we take the SIPAs from the major sport newspaper, *La Gazzetta dello Sport*, and add to the specification dummy variables on the journalist who made the SIPA. Our main conclusions are confirmed, both qualitatively and quantitatively.

**Figure 3:**
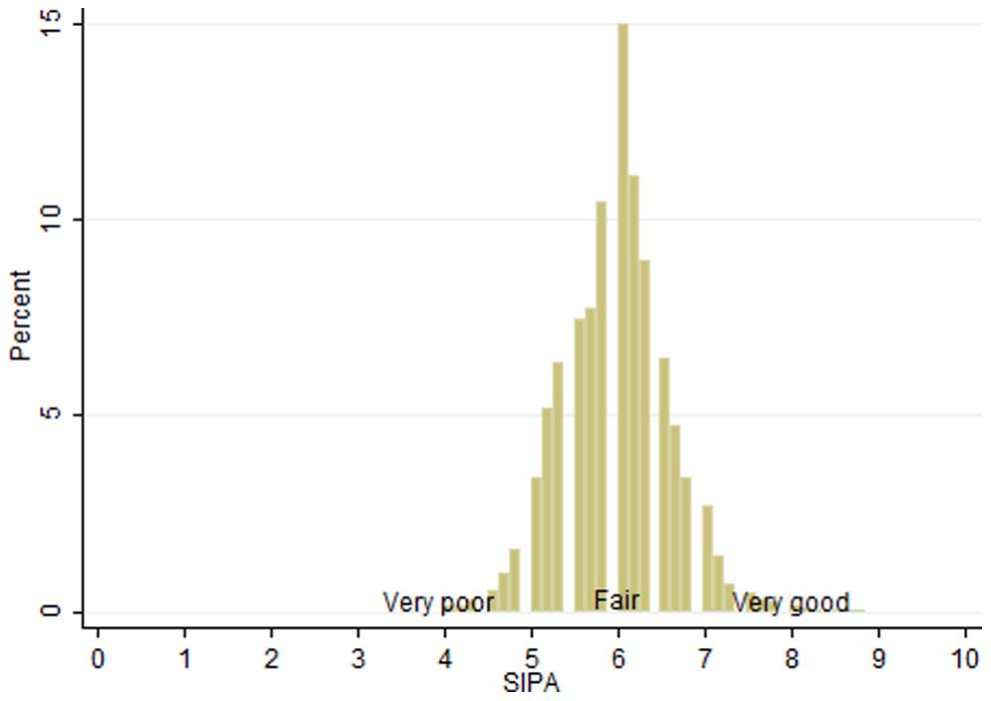
Distribution of individual SIPAs (8,226 observations).

Figure 3 plots the distribution of SIPAs in our sample. We see that SIPAs are concentrated between 4 and 9, with a peak around 6 (fair performance). Table 5 reports some summary statistics at the player level. First of all, we notice that SIPAs are generally higher when the team wins a match. However, low SIPAs are possible also in this case: players may indeed receive a SIPA of 4 even if their team wins the match. Moreover, the table lists some statistics about the main player's characteristics: his pay, his age, his past experience with the team and the first division, and his role (midfielder, forward, as opposed to goalkeeper or defender). We observe wide heterogeneity on these variables.

**Table 5:** Summary statistics, individual players (8,226 observations on 876 players).

Variable	Median	Mean	Std. dev.	Minimum	Maximum
**SIPA**					
If win	6.333	6.308	0.563	4	8.833
If draw	6	5.968	0.504	4	7.833
If defeat	5.667	5.631	0.531	4	7.833
OVERALL	6	5.966	0.611	4	8.833
**Individual variables**					
Pay (thousands of euros)	600	1029.200	1255.642	30	10500
Pay/average pay	0.920	0.997	0.496	0.010	4.348
New to the team	0	0.271	0.445	0	1
Years on the team	1	2.287	2.679	0	18
Years in first division	4	4.789	3.807	0	18
Age	27	27.429	3.954	17	41
Midfield role	0	0.399	0.490	0	1
Forward role	0	0.191	0.393	0	1

SIPAs show a weakly positive correlation with individual salaries (0.09) and team average pay (0.05), and a weakly negative correlation with pay dispersion (−0.05). It is also interesting to understand which “technology of production”—meant as a combination of individual SIPAs—determines team performance. If we regressed team performance over the minimum, mean, and maximum SIPAs of the team in the match (controlling for team, coach, match, opponent, and time characteristics), we would find all coefficients to be significant at 1%, suggesting that different technologies coexist. However, the average marginal effect of the mean SIPA is quantitatively much higher: 0.717, as opposed to −0.069 for the minimum SIPA and 0.129 for the maximum SIPA. This suggests that team performance depends on the individual effort of *all* players, more than on the effort of the best/worst ones.

The analysis in this section is meant to assist in understanding the link between individual performance and pay dispersion using an approach similar to our benchmark analysis. For this purpose we run a regression analysis, where the dependent variable is the individual SIPA, and the specification includes variables on the player (pay relative to the average pay, age, experience, and role), as well as the same variables used in Table 4. We consider ATMs as team members to construct our statistics. Table 6 shows the output from this regression, where we estimate the coefficients using a pooled ordinary least squares (OLS) method with player-clustered standard errors (column 1), a random-effect (RE) panel GLS method (column 2), or a fixed-effect (FE) panel OLS method (column 3); the latter method does not allow us to separate the effect of match-invariant variables from the player-specific effect. The “rho” coefficient reported in the bottom part of the table suggests that, in this context, it is important to consider player-specific effects. Moreover, the statistical tests comparing the three models, reported at the end of the table, suggest that it is advisable to use a panel method.

**Table 6:** Individual performance and pay dispersion (average marginal effects).

		(1)	(2)	(3)
Method:		Pooled OLS	RE GLS	FE OLS
Player:	Pay/average pay	0.105***	0.110***	−0.220
		(0.021)	(0.020)	(0.172)
	New to the team	−0.002	−0.004	
		(0.026)	(0.023)	
	Years on the team	0.014***	0.015***	
		(0.005)	(0.005)	
	Years in first division	−0.005	−0.005	
		(0.004)	(0.004)	
	Age	0.001	0.000	
		(0.003)	(0.003)	
	Midfield role	0.047**	0.050***	
		(0.019)	(0.019)	
	Forward role	−0.015	−0.014	
		(0.028)	(0.026)	
Pay:	Log(average pay)	0.082***	0.080***	−0.253
		(0.019)	(0.017)	(0.196)
	Log(pay dispersion index)	−0.064***	−0.080***	−0.139***
		(0.016)	(0.014)	(0.024)
Team:	Fraction of new players on the team	0.053	0.023	−0.007
		(0.073)	(0.071)	(0.115)
	Years on the team	−0.006	−0.012	−0.026
		(0.014)	(0.013)	(0.025)
	Years in first division	0.001	0.002	−0.007
		(0.010)	(0.010)	(0.018)
	Age	−0.009	−0.003	0.014
		(0.010)	(0.009)	(0.016)
Coach:	New to the team	−0.008	0.003	0.094
		(0.028)	(0.027)	(0.098)
	Replaced during the season	−0.016	0.012	0.134**
		(0.027)	(0.027)	(0.059)
	Years on the team	−0.011	−0.009	0.055*
		(0.014)	(0.013)	(0.033)
	Years in first division	0.007**	0.007**	−0.001
		(0.003)	(0.003)	(0.011)
	Age	0.001	0.002	0.004
		(0.002)	(0.002)	(0.007)
Match:	Injured players	−0.010**	−0.006	−0.000
		(0.005)	(0.004)	(0.005)
	Disqualified players	0.048***	0.056***	0.072***
		(0.011)	(0.011)	(0.012)
	Home play	0.144***	0.144***	0.140***
		(0.013)	(0.013)	(0.013)
Opponent:	Log(average pay)	−0.042***	−0.043***	−0.048***
		(0.014)	(0.013)	(0.013)
	Log(pay dispersion index)	0.055***	0.053***	0.053***
		(0.012)	(0.011)	(0.012)
	Fraction of new players on the team	0.035	0.058	0.120**
		(0.056)	(0.057)	(0.058)
	Years on the team	−0.001	0.003	0.011
		(0.010)	(0.010)	(0.010)
	Years in first division	0.004	0.005	0.009
		(0.006)	(0.006)	(0.007)
	Age	0.007	0.007	0.003
		(0.006)	(0.006)	(0.007)
	Constant	5.443***	5.217***	7.126***
		(0.308)	(0.276)	(1.521)
*+ time dummy variables on month and year of the match*				
	R^2^	0.041	0.040	0.000
	Rho coefficient		0.063	0.380
	Test pooled vs. panel		276.810	1.930
			[0.000]	[0.000]

*Note: 8,226 observations on 876 players (on average, 9.39 matches per player). The dependent variable is the average SIPA from three newspapers. Standard errors are given in parentheses; p values in brackets. In column 1 we report player-clustered standard errors*.

*p<0.1;

**p<0.05;

***p<0.01.

Our main findings are as follows. In columns (1) and (2), where we can estimate the effects of match-invariant variables, we find positive effects for individual pay, years of experience with the team, and the midfield role of the player (a core role in soccer). The direction of all of these effects is intuitive. Notice in particular that a high relative pay seems to work as an incentive on individual performance; this result is in line with, for instance, the results of Pfeffer and Langton (1993) [41]. However, giving a disproportionately high pay to some is not necessarily an effective strategy. In fact, it may give rise to high pay dispersion, and in Table 6 we consistently find a negative effect for the team pay dispersion. In addition, we find positive effects for playing at home, number of disqualified players, and the opponent's pay dispersion, and a negative effect for the opponent's average pay.

In Section B.5 of the supplementary material (available upon request), we repeat the same analysis, adding into the specification variables that consider whether the player is a “superstar” (when he earns at least two times the average pay in the team) or a “regular player” (one of the 11 most frequent players in the first month of the season), alone and interacting with pay dispersion. Interestingly, we find that SIPA increases with regular players, and responds more negatively to pay dispersion among superstars. In particular the first result suggests that infrequent players, when they have “all eyes on them” during the match, are not able to perform as well as the regular players for whom they substitute.

**Figure 4:**
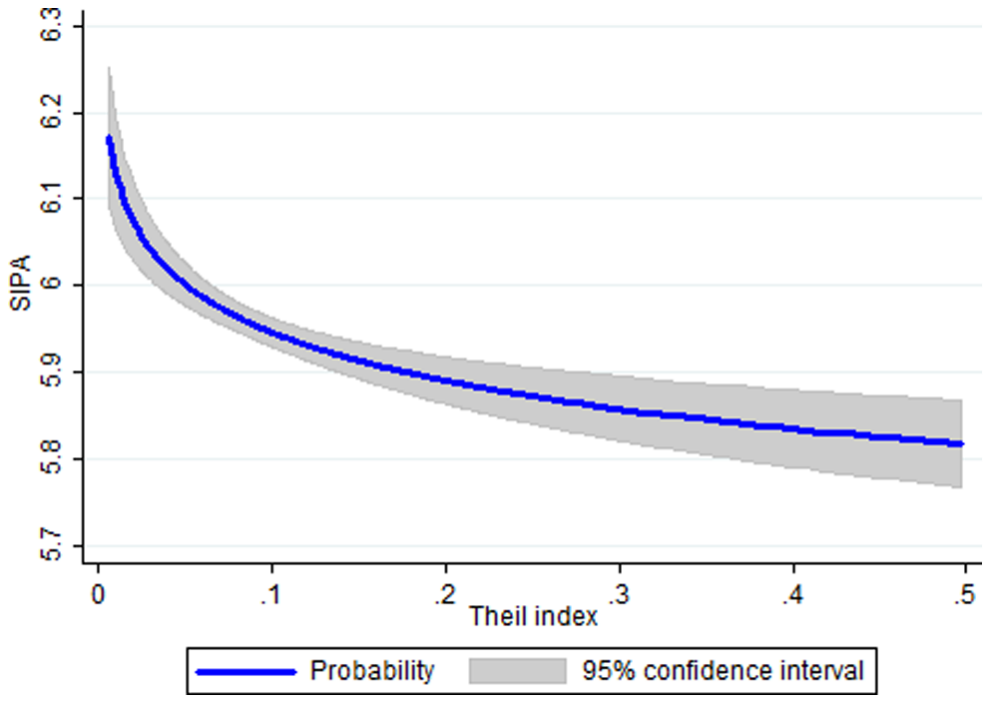
Predicted SIPA, by pay dispersion. Note: Predictions are based on the average explanatory variables and the parameter estimates from Table 6, column (3).

Figure 4 reports the predicted SIPA conditional on pay dispersion and the average explanatory variables, using the estimates from column (2) of Table 6. We focus on this column, rather than column (3), because it shows a lower effect of pay dispersion (−0.08 instead of −0.14), and overall it provides more convincing estimates—in particular, because it shows significant effects as a result of the players' and team salaries. As its counterpart for team performance (panel [a] of Figure 2), the figure shows that the SIPA is the highest when there is no pay dispersion at all. This suggests that pay dispersion has a detrimental effect not just on team performance, but that it also negatively impacts individual performance.

To interpret this figure, we return to our previous example with the team manager. Suppose the manager has to choose whether to increase or decrease the current pay dispersion (where 1 player earns 2.5 times the average income, and each of the 10 remaining players earns 0.85 times the average income). The outcome of this choice is not trivial, because varying the distribution of pays affects not only pay dispersion, but also the average pay and the players' pay, which in turn have different implications on individual performance. To keep the situation simple, let us say that the manager has a budget balance and he considers two alternatives that do not alter average pay: in plan A, the top player earns three times the average pay, and each remaining player earns 0.8 times the average pay; in plan B, the top player earns two times the average pay, and each remaining player earns 0.9 times the average pay. The corresponding Theil index goes from an initial level of *T* = 0.083 to either *T* = 0.137 in plan A or *T* = 0.040 in plan B.

We know from Table 6 that an increase in player's pay has a positive effect on individual performance, while an increase in pay dispersion has a negative effect. As a result, the direction of the effect on the top player is unclear *a priori*, while we already know that in plan A the performance of the lower-paid players will fall, and in plan B their performance will rise. With these numbers we find that, in plan A the SIPA of the top player will rise by 0.015 points, whereas the SIPA of each other player will fall by 0.046 points. In plan B, the SIPA of the top player will rise by 0.003 points (notwithstanding a reduction of his pay), while the SIPA of each other player will rise by 0.064 points. All in all, the effect on the top player is lower than that on the other players. Considered along with the fact that there is just one top player, but 10 other players, this suggests that plan B is preferable, because it increases the average SIPA by 0.058 points; in contrast, plan A reduces the average SIPA by 0.04 points.

## Conclusions

Relatively little is known about how the introduction of dispersed pay in teams influences team performance. However, teams are becoming increasingly widespread in organizations [42]–[43]. The same is true for performance-contingent pay on the individual level [44]. In fact, firms introduce dispersed pay in teams [45]–[46], but the effect of this managerial intervention on team performance has thus far been unclear. The broader literature on pay dispersion in organizations has resulted in very different, even contradictory, findings. In fact, the extant research suggests that the effect of pay dispersion on organizational performance can be positive, null, or negative [47].

In this study we collected and analyzed a unique dataset of matches played during two seasons of the men's major soccer league in Italy. This unique dataset allows us to measure the effect of pay dispersion according to different definitions of team. This peculiarity of our dataset is the crucial element that can explain the conflicting evidence. Indeed, we also find positive, null, and negative effects of pay dispersion on team performance, using the *same data* but *different definitions of team*. However, when we take the narrowest definition of a team—considering only the members who actually took part in the task and how long they played—pay dispersion has a detrimental impact on team performance: doubling pay dispersion decreases by 6% the probability of winning a match. This result is consistent with several robustness checks.

This negative effect of pay dispersion on team performance may be the reason why salaries are usually kept secret within a firm [48]. Employees do not like to earn less than their coworkers; as a result, pay dispersion can decrease cooperation within the team and it may affect individual performance. We investigate this issue in our environment by looking at the number of passes within a match and the (subjective) individual performance assessments reported by the three most important Italian sports newspapers. Our results show that higher pay dispersion has a detrimental impact on individual evaluations, whereas it does not have a significant effect on cooperation.

Our results hold for any level of pay given the dimensionless nature of our pay dispersion index. Therefore, the external validity of our analysis goes beyond this specific sports environment. Actually, the fact that in the sport environment salaries can be high is a strong feature of our dataset, because it allows us to study a large variety of pay dispersions.

One limitation of our analysis is that the association between pay dispersion and team performance is really driven by underlying differences in players skills, abilities and talents and that such differences cause both pay dispersion and team performance. We cannot resolve this issue, given our data. The proper way to address it arguably is by means of an experimental research design that allows the experimentalist to control talent-related as well as pay-related variables. We hope our study will inspire future research into this issue.

## References

[1] Lawler EE (1990) Strategic pay: Aligning organizational strategies and pay systems. San Francisco, CA: Jossey-Bass.

[2] Shaw JD, Gupta N, Delery JE (2002) Pay dispersion and workforce performance: Moderating effects of incentives and interdependence. Strategic Management Journal 23: 491–512.

[3] Milkovich GT, Newman JM (1996) Compensation. (5th ed.) Homewood IL: Irwin: 45.

[4] Jirjahn W, Kraft K (2007) Intra-firm wage dispersion and firm performance – Is there a uniform relationship? Kyklos 60 2: 231–253.

[5] Trevor CO, Reilly G, Gerhart B (2012) Reconsidering pay dispersion's effect on the performance of interdependent work: Reconciling sorting and pay inequality. Academy of Management Journal 55: 585–610.

[6] Martin J (1981) Relative deprivation: A theory of distributive injustice for an era of shrinking resources. In LL Cummings, BM Staw (eds.) Research in Organizational Behavior 3. Greenwich, CT: JAI Press.

[7] Lazear EP, Rosen S (1981) Rank-order tournaments as optimum labor contracts. The Journal of Political Economy 89: 841–864.

[8] Ramaswamy R, Rowthorn RE (1991) Efficiency pays and pays dispersion. Economica 58: 501–14.

[9] Aime F, Meyer CJ, Humphrey SE (2010) Legitimacy of team rewards: Analyzing legitimacy as a condition for the effectiveness of a team incentive designs. Journal of Business Research 61: 60–66.

[10] Milgrom P, Roberts J (1992) Economics, organization, and management, upper saddle river. NJ: Prentice Hall.

[11] Ramaswamy R, Rowthorn RE (1991) Efficiency pays and pays dispersion. Economica 58: 501–14.

[12] Avrutin BM, Sommers PM (2007) Work incentives and salary distributions in Major League Baseball. Atlantic Economic Journal 35: 509–510.

[13] Berri DJ, Jewell TR (2004) Pay inequality and firm performance: Professional basketball's natural experiment. Atlantic Economic Journal 32: 130–139.

[14] Katayama H, Nuch H (2011) A game-level analysis of salary dispersion and team performance in the National Basketball Association. Applied Economics 43: 1193–1207.

[15] Lazear EP, Shaw KL (2007) Personnel economics: the economist's view of human resources, Journal of Economic Perspectives. 21: 91–114.

[16] Stewart GL (2006) A meta-analytic review of relationships between team design features and team performance. Journal of Management 32: 29–54.

[17] LePine AL, Piccolo RF, Jackson CL, Mathieu JE, Saul JR (2008) A meta-analysis of teamwork processes: Tests of a multidimensional model and relationships with team effectiveness criteria. Personnel Psychology 61: 273–307.

[18] Park G, Spitzmuller M, DeShon RP (2013) Advancing our understanding of Team motivation – Integrating conceptual approaches and content areas. Journal of Management 39 5: 1339–1379.

[19] Hamilton B, Nickerson JA, Owan H (2003) Team incentives and worker heterogeneity: An empirical analysis of the impact of teams on productivity and participation. Journal of Political Economy 111: 465–497.

[20] Guzzo RA, Dickson MW (1996) Teams in organizations: Recent research on performance and effectiveness. Annual Review of Psychology 47 1: 307–338. 10.1146/annurev.psych.47.1.307 15012484

[21] Zenger T, Hesterly W (1997) The disaggregation of corporations: Selective intervention, high-powered incentives, and molecular units. Organization Science 8 3: 209–222.

[22] Davis SJ, Haltiwanger J (1991) Wage dispersion between and within U.S. manufacturing plants. In Baily M & Winston C eds., Brookings papers on Economic Activity, Microeconomics: 115–200.

[23] Zenger T, Hesterly W (1997) The disaggregation of corporations: Selective intervention, high-powered incentives, and molecular units. Organization Science 8 3: 209–222.

[24] Aime F, Meyer CJ, Humphrey SE (2010) Legitimacy of team rewards: Analyzing legitimacy as a condition for the effectiveness of a team incentive designs. Journal of Business Research 61: 60–66.

[25] Fredrickson JW, Davis-Blake A, Sanders W (2010) Sharing the wealth: social comparisons and pay dispersion in the CEO's top team. Strategic Management Journal 31: 1031–1053.

[26] Bloom M (1999) The performance effects of pay dispersion on individuals and organizations. Academy of Management Journal 42: 25–40.

[27] Becker BE, Huselid MA (1992) The incentive effects of tournament compensation systems. Administrative Science Quarterly 37: 336–350.

[28] Ehrenberg RG, Bognanno ML (1990) Do tournaments have incentive effects? The Journal of Political Economy 98: 1307–1324.

[29] Marchand JT, Smeeding TM, Torrey BB (2006) Salary distribution and performance: Evidence from the National Hockey League, unpublished manuscript, Department of Economics and Center for Policy Research, Maxwell School of Citizenship and Public Affairs, Syracuse University and Population Reference Bureau.

[30] Bloom M (1999) The performance effects of pay dispersion on individuals and organizations. Academy of Management Journal 42: 25–40.

[31] Depken CA (2000) Pay disparity and team productivity: Evidence from Major League Baseball. Economics Letters 67: 87–92.

[32] Jane W (2010) Raising salary or redistributing it: A panel analysis of major league baseball. Economics Letters 107: 297–299.

[33] Wiseman F, Chatterjee S (2003) Team payroll and team performance in Major League Baseball: 1985–2002. Economics Bulletin 1: 1–10.

[34] Avrutin BM, Sommers PM (2007) Work incentives and salary distributions in Major League Baseball. Atlantic Economic Journal 35: 509–510.

[35] Berri DJ, Jewell TR (2004) Pay inequality and firm performance: Professional basketball's natural experiment. Atlantic Economic Journal 32: 130–139.

[36] Katayama H, Nuch H (2011) A game-level analysis of salary dispersion and team performance in the National Basketball Association. Applied Economics 43: 1193–1207.

[37] Pfeffer J, Langton N (1993) The effect of wage dispersion on satisfaction, productivity, and working collaboratively: Evidence from college and university faculty. Administrative Science Quarterly 38: 382–407.

[38] Deloitte (2011) Annual review of football finance 2011. Manchester, UK: Deloitte Touche Tohmatsu Limited.

[39] Kahn LM (2000) The sports business as a labor market laboratory. Journal of Economic Perspectives 14: 75–94.

[40] Scully GW (1974) Pay and performance in Major League Baseball. The American Economic Review 64: 915–930.

[41] Pfeffer J, Langton N (1993) The effect of wage dispersion on satisfaction, productivity, and working collaboratively: Evidence from college and university faculty. Administrative Science Quarterly 38: 382–407.

[42] Lazear EP, Shaw KL (2007) Personnel economics: the economist's view of human resources. Journal of Economic Perspectives 21: 91–114.

[43] Stewart GL (2006) A meta-analytic review of relationships between team design features and team performance. Journal of Management 32: 29–54.

[44] Davis SJ, Haltiwanger J (1991) Wage dispersion between and within U.S. manufacturing plants. In Baily M & Winston C eds., Brookings papers on Economic Activity. Microeconomics: 115–200.

[45] Bloom M (1999) The Performance effects of pay dispersion on individuals and organizations. Academy of Management Journal 42: 25–40.

[46] Fredrickson JW, Davis-Blake A, Sanders W (2010) Sharing the wealth: social comparisons and pay dispersion in the CEO's top team. Strategic Management Journal 31: 1031–1053.

[47] Jirjahn W, Kraft K (2007) Intra-firm wage dispersion and firm performance – Is there a uniform relationship? Kyklos 60 2: 231–253.

[48] Lawler EE (1990) Strategic Pay: Aligning organizational strategies and pay systems. San Francisco, CA: Jossey-Bass. 238–42.

